# Molecular characterization of breast cancer needle core biopsy specimens by the 21‐gene Breast Recurrence Score test

**DOI:** 10.1002/jso.26050

**Published:** 2020-06-04

**Authors:** Debbie M. Jakubowski, Helen Bailey, John Abran, Andrea Blacklock, Nancy Ciau, Carolyn Mies, Vivian Tan, Rebekah Young, Anna Lau, Frederick L. Baehner

**Affiliations:** ^1^ Exact Sciences Corporation Redwood City California

**Keywords:** core needle biopsy, COVID‐19 pandemic, Oncotype DX, Recurrence Score result

## Abstract

**Background and Objective:**

Recent COVID‐19 pandemic guidelines recommend genomic assessment of core biopsies to help guide treatment decisions in estrogen receptor (ER)‐positive early‐stage breast cancer. Herein we characterize biopsy and excisional breast cancer specimens submitted for 21‐gene testing.

**Methods:**

US samples submitted to Genomic Health for 21‐gene testing (01/2004‐04/2020) were assessed by pathologists and analyzed by a standardized quantitative reverse transcription‐polymerase chain reaction. Predefined cutoffs were: *ESR1* (positive ≥6.5), *PGR* (positive ≥5.5), and *ERBB2* (negative <10.7). ER status by immunohistochemistry (IHC) and lymph node status were determined locally. Median and interquartile range were reported for continuous variables, and total and percent for categorical variables. Distributions were assessed overall, by age, and by nodal involvement.

**Results:**

Of 919 701 samples analyzed, 13% were biopsies and 87% were excisions. Initial assay success rates were 94.5% (biopsies) and 97.3% (excisions). ER IHC concordance with central *ESR1* was 96.8% (biopsies) and 97.6% (excisions). Biopsy and excisional medians were: Recurrence Score results 16 (each); *ESR1* 10.2 (each); *PGR* 7.7 and 7.6; *ERBB2* 9.4 and 9.2, respectively.

**Conclusions:**

Biopsy submissions for 21‐gene testing are common and consistently generate results that are very similar to the experience with excisions. The 21‐gene test can be performed reliably on core biopsies.

## 1 INTRODUCTION

The COVID‐19 pandemic poses unprecedented challenges for patients, clinicians, and healthcare systems. In response to the pandemic, physicians are modifying breast cancer care, prioritizing patients by treatment urgency to minimize exposure risk and conserve resources without significantly compromising long‐term outcomes for individual patients. Neoadjuvant treatment enables delayed surgery and is well established for all breast cancer subtypes.

The recently revised COVID‐19 pandemic guidelines recommend consideration of neoadjuvant endocrine treatment for patients with early‐stage, estrogen receptor (ER)‐positive, human epidermal growth factor 2 (HER2)‐negative tumors in whom surgery can be deferred.^1^, ^2^, ^3^, ^4^ For patients with stage 1 or limited stage 2 disease (including those with N1 nodal involvement), the following features derived from the diagnostic needle core biopsy can assist in determining who will likely not benefit substantially from neoadjuvant or adjuvant chemotherapy and may receive endocrine therapy alone: low‐intermediate grade tumors, lobular histology, and low‐risk genomic assay results.^2^

Guidelines have long recommended use of core biopsy samples for assessment of receptor status by immunohistochemistry (IHC) and fluorescent in situ hybridization (FISH) despite concordance rates below 100% between paired core biopsy and subsequent excisional specimens.^5^, ^6^, ^7^, ^8^, ^9^, ^10^, ^11^, ^12^ In addition to receptor status, needle core biopsies provide clinicians with foundational information, including histologic subtype and tumor grade, to inform neoadjuvant treatment decisions, information that may or may not correlate with subsequent excisional samples.^13^, ^14^

The 21‐gene test (Oncotype DX Breast Recurrence Score® test; Exact Sciences Corp, Redwood City, CA) is a validated prognostic tool and predictor of adjuvant chemotherapy benefit in patients with ER+, HER2−, node‐negative, or node‐positive early breast cancer who receive 5 years of hormonal therapy.^15^, ^16^, ^17^, ^18^, ^19^, ^20^, ^21^ The 21‐gene test is also validated to predict response to neoadjuvant endocrine therapy in patients with ER+, HER2−, clinically node‐negative breast cancer.^22^ Patients with low Recurrence Score® (RS) results have an increased likelihood of clinical response and breast‐conserving surgery with neoadjuvant endocrine therapy.^22^, ^23^, ^24^ Additionally, multiple studies have shown an association between RS group and response to neoadjuvant chemotherapy: patients with higher RS results are more likely to achieve a pathological complete response (pCR) to neoadjuvant chemotherapy than those with lower results.^25^, ^26^, ^27^, ^28^, ^29^, ^30^, ^31^, ^32^

Here we characterize by pathology review and by quantitative reverse transcription‐polymerase chain reaction (RT‐PCR) analysis the biopsy and excisional breast cancer specimens from clinical practices in the United States submitted to the Genomic Health (now Exact Sciences) Clinical Laboratory for 21‐gene testing.

## 2 METHODS

Domestic invasive breast cancer tumor specimens examined in the Genomic Health (now Exact Sciences) Clinical Laboratory from January 2004 through April 2020 were included in these analyses. The 21‐gene Oncotype DX Breast Recurrence Score algorithm, based on quantitative expression of 16 cancer‐related genes and five reference genes, has been previously described.^15^ Briefly, gene expression is measured in triplicate using quantitative RT‐PCR from formalin‐fixed, paraffin‐embedded tumor tissue assessed microscopically and microdissected when tumor tissue is less than 50% or there are contaminants such as biopsy cavities present. RS results range from 0 to 100, where a higher result indicates an increased risk of recurrence. Sample type, RNA yield (ng/µL), quantitative single‐gene expression, and RS results are captured.

To protect patient identification, dates of birth, dates of specimen collection, and dates of specimen receipt at the laboratory were not provided to the study team. Instead, only patient age (calculated by the Data Management team as date of specimen collection minus date of birth) were provided to the study team. Due to data entry errors or missing data, there are instances of age falling outside of realistic ranges. To remove any implausible ages, data were trimmed at the 0.05th percentiles of captured age. Eligible records were those that were not missing key variables: specimen type, age, and genetic data.

Lymph node status was determined by local review and patients were categorized as having node‐negative (N0), micrometastatic (N1mi), node‐positive (N+), or unknown nodal involvement. Any record missing specimen type (biopsy vs excisional sample), RNA yield, RS result, or patient age was excluded. Classifications of *ESR1* status were based on the pre‐established cycle threshold (C_t_) of 6.5.^33^

Because of the large study sample size, all analyses were descriptive, as even small differences between groups are expected to be statistically significant but potentially not clinically meaningful. Median and interquartile range (IQR) were reported for continuous variables, and total and percent for categorical variables. The distributions of quantitative single‐gene, gene group, and RS results were visually presented using ridge plots by specimen type. Distributions were assessed overall, by age (<50 vs ≥50 years), and by nodal involvement (N0, N1mi, N+, unknown). Categorical ER status (positive or negative) by IHC is captured on the requisition form, and when ER status is flagged as negative by RT‐PCR, the sample is reviewed by the internal pathology team. Concordance of categorical local ER by IHC and central *ESR1* was calculated by specimen type. Analyses were performed in R (R Foundation for Statistical Computing, Vienna, Austria) and SAS v9.4 (SAS Institute Inc, Cary, NC). Institutional Review Board reviewed and approved the study plan, finding it to have met the requirements for a waiver of consent.

## 3 RESULTS

Between January 2004 and April 2020, a total of 1 048 881 invasive breast cancer specimens were examined in the Exact Sciences (formerly Genomic Health) Clinical Laboratory. Of these, 972 673 were processed. The first‐pass success rates varied by specimen type: 94.5% of biopsies generated patient reports with 3.5% biopsies failing pathology review and 2.0% failing in the laboratory; 97.3% of excisional samples generated patient reports with 1.9% of excisional samples failing pathology review and 0.8% failing in the laboratory (Figure S1). The final success rates for each sample type were higher. RNA yield was on average lower in biopsy than in excisional samples: median (IQR) yields of 1391 ng/µL (765‐2507 ng/µL) from biopsies and 2605 ng/µL (1345‐5013 ng/µL) from excisions.

The final analysis cohort that met all eligibility criteria comprised 919 701 specimens, of which 13% were biopsy samples, and 87% excisional samples. Median patient age was 61 years (IQR: 52‐68 years) for biopsy samples and 60 years (IQR: 51‐67 years) for excisional samples; 19% of patients with biopsy and 20% of patients with excisional samples were younger than 50 years of age. Nodal status was unknown in 21% of biopsy and 4% of excisional samples. Among those with known nodal involvement, 85%, 3%, and 12% of biopsies and 84%, 5%, and 11% of excisional samples were categorized as N0, N1mi, and N+, respectively. Concordance in categorical ER status was 96.8% for biopsies and 97.6% for excisional samples (Table S1). Among those with a successful RS result, the *ESR1* positive rate among biopsy tests was 97.1% and differed with local ER IHC assessment in 2.9% of cases, while the *ESR1* positive rate among excisional sample tests was 97.9% and differed in 2.1% of cases.

**Figure 1:**
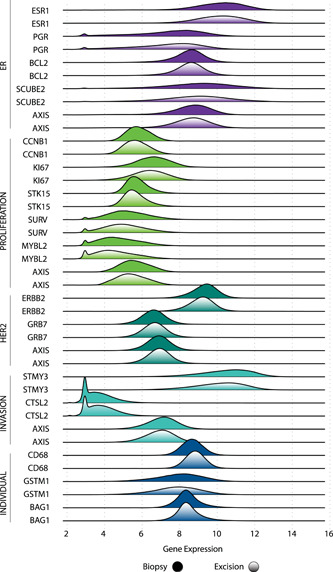
Quantitative single‐gene expression and gene group score distributions, by specimen type [Color figure can be viewed at wileyonlinelibrary.com]

**Figure 2:**
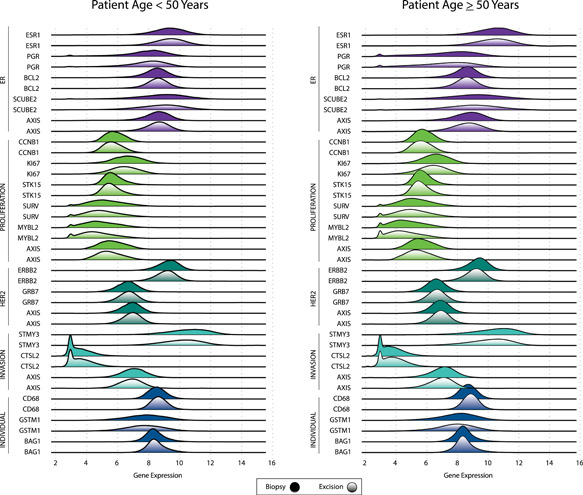
Quantitative single‐gene expression and gene group score distributions, by specimen type and age group [Color figure can be viewed at wileyonlinelibrary.com]

**Figure 3:**
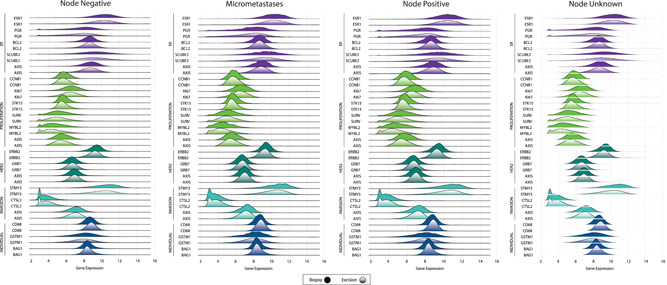
Quantitative single‐gene expression and gene group score distributions, by specimen type and nodal status [Color figure can be viewed at wileyonlinelibrary.com]

Overall, quantitative single‐gene expression and gene group score distributions were similar between biopsy and excisional data (Figure 1), a result that remained consistent when assessed by age (Figure 2) and nodal involvement (Figure 3). Median (IQR) quantitative expression of *ESR1* was 10.2 (9.3‐11.0) in core biopsies and 10.2 (9.3‐11.0) in excisional samples. Median (IQR) quantitative expression of *PGR* was 7.7 (6.3‐8.7) in biopsies and 7.6 (6.2‐8.6) in excisional samples. Median (IQR) quantitative expression of *ERBB2* was 9.4 (8.9‐9.8) in biopsies and 9.2 (8.7‐9.6) in excisional samples. Median (IQR) proliferation gene group score was 5.6 (5.1‐6.2) in biopsies and 5.4 (4.9‐6.0) in excisional samples.

**Figure 4:**
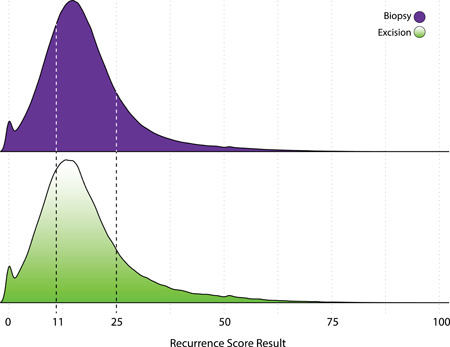
Distribution of Recurrence Score results, by specimen type [Color figure can be viewed at wileyonlinelibrary.com]

The distribution of RS results was similar between core biopsy and excisional specimens, with median (IQR) of 16 (10‐22) in biopsies and 16 (11‐22) in excisions (Figure 4). The distributions of RS results by age (Table 1 and Figure S2) and by nodal involvement (Figure S3) had similar findings. Among N0 samples, 82.5% of core biopsies and 83.2% of excisions had an RS result of 0 to 25. Among N1mi and N+ samples combined, 85.1% of biopsies and 86.3% of excisions had an RS result of 0 to 25. Among younger patients (age <50 years) with N0 disease, the distribution of RS groups (RS: 0‐10, 11‐15, 16‐20, 21‐25, and 26‐100) was similar between biopsy and excisional samples (Figure S4).

**Table 1:** Distribution of Recurrence Score group by specimen type (biopsy vs excision) and age group (<50 vs ≥50 years)

		Recurrence Score group				
		RS 0‐10	RS 11‐15	RS 16‐20	RS 21‐25	RS 26‐100
Age <50 y	Biopsy (n = 22 926)	17.6%	26.2%	23.2%	12.9%	20.0%
	Excision (n = 163 912)	18.3%	27.9%	25.2%	13.1%	15.4%
Age ≥50 y	Biopsy (n = 96 789)	27.2%	24.1%	19.4%	11.6%	17.7%
	Excision (n = 636 074)	23.0%	24.3%	22.4%	13.6%	16.7%

Abbreviation: RS, Recurrence Score result.

## 4 DISCUSSION

Analyses of the Exact Sciences Clinical Laboratory experience from nearly a million breast cancer samples show a high degree of success in obtaining results from core biopsy samples submitted to the laboratory. These analyses show a high degree of concordance between locally determined ER IHC status and central *ESR1* quantitative gene result. The distributions of RS results, the quantitative single‐gene results for *ESR1, PGR*, and *ERBB2*, and other cancer‐related gene results obtained from core biopsy and excisional samples are highly overlapping, overall and when analyzed by patient age group (<50 vs ≥50 years) or by nodal status (N0, N1mi, N+, or unknown).

The similarity of the results highlights the lack of bias in results derived from core biopsy vs excisional samples. As such, these important data supplement reports of the analytical validation of the 21‐gene test,^34^ including the concordance of *ESR1, PGR*, and *ERBB2* assessment by the 21‐gene test and ER, PR, and HER2 assessment by IHC or FISH.^35^, ^36^ These consistent results support the extensive clinical validation experience of the 21‐gene assay in both the adjuvant and neoadjuvant setting, including studies showing the correlation of RS results derived from core biopsies with response to neoadjuvant systemic therapy.^22^, ^23^, ^24^, ^25^, ^26^, ^27^, ^28^, ^29^, ^30^, ^31^, ^32^ Importantly, our results support guidelines recommending determination of the 21‐gene results from core biopsy samples for use in clinical treatment decision making.

In response to the COVID‐19 pandemic, guidelines recommend that physicians use information derived from core biopsy samples to assist in planning for breast cancer care. The intent is to identify candidates for neoadjuvant endocrine therapy for whom surgery may be delayed without significantly compromising long‐term outcomes, to minimize coronavirus exposure risk and immunosuppression. For patients with ER+, HER2− breast cancer, this recommendation is supported by a meta‐analysis demonstrating similar success in achieving breast‐conserving surgery, although lower rates of pCR, with neoadjuvant endocrine therapy vs neoadjuvant chemotherapy.^37^ Other studies of endocrine therapy with or without surgery show no difference in survival for the first 3 years, suggesting no adverse short‐term outcomes with endocrine therapy but delayed surgery.^38^, ^39^ Use of the core biopsy is foundational to treatment planning, to identify patients with early‐stage ER+ HER2− breast cancer and low‐intermediate grade tumors, lobular histology, or low‐risk genomic assays results for whom neoadjuvant endocrine treatment can be administered when surgery is deferred.^1^, ^2^, ^3^, ^4^

There is extensive literature examining the concordance between paired core biopsy and excisional samples underlying the recommendations of breast cancer clinical guidelines to use core biopsies for routine assessment of ER, PR, and HER2 receptor status, tumor grade, and histologic subtype.^5^, ^6^, ^7^, ^8^ In a contemporary, comprehensive meta‐analysis of 21 studies evaluating hormone receptor status in paired needle core biopsy and excisional samples with no intervening treatment, concordance was 92.8% (*κ* = 0.78) for ER status (N = 2450 pairs) and 85.2% (*κ* = 0.66) for PR status (N = 2448 pairs).^9^ In another recent study of 1219 paired needle core biopsy and excisional samples with no intervening treatment, the overall agreement between needle core biopsy and excisional samples was 97.1% for ER (*κ* = 0.906), 95.0% for PR (*κ* = 0.877), and 84.6% for HER2 (*κ* = 0.672).^10^ A clinically similar degree of concordance has been reported for the 21‐gene assay using quantitative RT‐PCR by Stull et al.^40^ In 24 patients with paired needle core biopsy and excisional samples who had no intervening treatment, there was 100% concordance between *ESR1* positivity and negativity using the pre‐established cutoff of ≥6.5 C_t_, with a correlation coefficient of 0.84 (95% confidence interval [CI]: 0.65‐0.93) when assessed as a continuous variable, and 100% concordance between *ERBB2* positivity and negativity using the pre‐established cutoffs of <10.7 C_t_ for negativity and ≥11.5 C_t_ for positivity, with a correlation coefficient of 0.82 (95% CI: 0.61‐0.92) when assessed as a continuous variable. Concordance of *PGR* positivity and negativity was lower at 88% (two core biopsies were *PGR*‐positive but negative in the paired excisions, and one core biopsy was *PGR*‐negative but positive in the paired excision), although the correlation coefficient was 0.83 (95% CI: 0.64‐0.92) when assessed as a continuous variable.^40^ The present study results further support the body of evidence supporting the use of core biopsies for molecular testing: (a) the receptor status results show that the concordance between local ER by IHC and central *ESR1* by RT‐PCR is high for core biopsies (97.1%) and excisional samples (97.9%); and (b) there is no apparent bias between core biopsy and excisional samples when assessed for *ESR1, PGR*, and *ERBB2*, based on median (IQR) quantitative expression values that are almost identical.

Histologic grade is a strong prognostic and predictive factor in ER+, HER2− early‐stage breast cancer.^41^, ^42^, ^43^, ^44^ Histologic grade stratifies patients into two categories: those with low‐grade tumors characterized by low proliferation/lower mitotic index who have a better prognosis, and those with high‐grade tumors characterized by high proliferation/high mitotic index with a poorer prognosis. Patients in the latter category often merit more aggressive treatment. Histologic grade derived from the needle core biopsy is a central determinant of neoadjuvant treatment planning. Studies of the concordance of histologic grade between paired core biopsy and excisional samples have shown lower concordance rates, generally under 80%.^11^, ^12^, ^14^ Among discordant cases, there was a bias toward underestimation of the histologic grade in the core biopsy vs the paired excisional samples by one grade level largely due to an underestimation ofmitotic index.^11^, ^12^, ^14^ In contrast to conventional methods of microscopic assessment of nuclear atypia and mitotic figure counting, the 21‐gene assay captures tumor proliferative activity by the quantitation of 5 proliferation genes that include *MKI67*, the gene for Ki67, and constitute the “proliferation gene axis.”^16^ The five proliferation genes are assessed as continuous covariates, and their expression average is integrated into the RS result when the group result is at or above the threshold of 6.5 C_t_. Where histologic assessment of mitotic index may differ between matched core biopsy and excisional samples, results for the proliferation gene axis by quantitative RT‐PCR did not significantly differ, as shown in the study by Stull et al.^40^ The present study extends this observation by demonstrating that the median (IQR) values for both the continuous and thresholded proliferation gene axis do not meaningfully differ between core biopsy and excisional specimens.

The RS result derived from paired core biopsy and excisional samples has been shown to be highly concordant when assessed as either categorical risk groups or continuous results (correlation coefficient 0.83; mean difference of 4.1 [95% CI: 1.7, 6.4] RS units between paired samples).^40^ The present study contributes to these data by showing that the distributions of RS results are very similar between populations treated according to core biopsies and excisional samples (median RS result of 16 for both sample types). Of significant clinical relevance for neoadjuvant treatment planning is that approximately 82% to 83% of N0 biopsy and excisional samples and 85% to 86% of N1mi/N+ biopsy and excisional samples in the present study had an RS result of 0 to 25. Among younger patients (age <50 years) with N0 disease, the distribution of RS groups (RS: 0‐10, 11‐15, 16‐20, 21‐25, and 26‐100) was similar between biopsy and excisional samples. RS subsets are clinically relevant, since some younger patients in TAILORx derived benefit from adjuvant chemotherapy at lower RS results.^20^

This report represents the largest study to date evaluating the consistency in molecular characteristics between biopsy and excisional specimens from a large central laboratory. Strengths of this study include the size of the study, with over 970 000 breast cancer specimens, of which more than 130 000 were biopsy samples (Figure S1). Additionally, the 21‐gene test is a standardized molecular assay that has been extensively clinically validated, including in TAILORx and in large population‐based analyses of N0 and N+ breast cancer.^15^, ^16^, ^17^, ^18^, ^19^, ^20^, ^21^, ^45^, ^46^, ^47^, ^48^ The analysis is limited by the fact that the biopsy and excisional samples were unmatched (ie, not from the same patients), so concordance per se between paired samples cannot be determined.

## 5 CONCLUSIONS

Breast cancer clinicians depend on needle core biopsy tumor tissue assessment for histologic features and receptor status to guide neoadjuvant treatment decisions. The data presented in these analyses demonstrate that determination of 21‐gene assay results from needle core biopsy specimens is reliable, with results that closely parallel those obtained from excisional specimens. The present study and the wider body of analytical and clinical evidence for the RS test support guideline recommendations for determination of the RS results from core biopsies to assist in neoadjuvant or adjuvant treatment decisions.

## CONFLICT OF INTERESTS

All authors declare that they are current employees of and own stock in Exact Sciences Corp.

## SYNOPSIS

Among 919 701 breast cancer specimens (13% biopsies, 87% excisions) submitted for 21‐gene testing, concordance between ER status by local immunohistochemistry and *ESR1* expression by central RT‐PCR was 96.8% (biopsies) and 97.6% (excisions). Biopsy and excisional medians were: RS results 16 (each); *ESR1* 10.2 (each); *PGR* 7.7 and 7.6; *ERBB2* 9.4 and 9.2, respectively. The 21‐gene test can be performed reliably on needle core biopsies and excisional samples.

## Supporting information

Supporting information

## Data Availability

The datasets generated for and analyzed in the current study are not publicly available because they contain patient data and proprietary information. Aggregated data generated for and analyzed in the current study are included in this article.
