# Supplementary material for: Molecular characterization of breast cancer needle core biopsy specimens by the 21‐gene Breast Recurrence Score test
Source: J Surg Oncol. 2020 Jun 4;122(4):611–8. doi: 10.1002/jso.26050 (PMC7496790; doi:10.1002/jso.26050)

Supplementary material

Supplementary Table 1. Concordance of ER status as measured by IHC and RT-PCR.

		ER by RT-PCR		Concordance
	ER by IHC†	Negative	Positive	
Biopsies	Negative	1,105*	449	96.8%
	Positive	3,412	115,376	
Excisions	Negative	3,986*	2,690	97.6%
	Positive	16,453	777,368	

*Samples found to be ER− by both IHC and RT-PCR are failed under the Clinical Laboratory pathology review.

†IHC data were unavailable for 3,953 specimens.

ER, estrogen receptor; IHC, immunohistochemistry; RT-PCR, reverse transcription polymerase chain polymerase.

Supplementary Figure 1. Initial laboratory failure rates, by specimen type (among samples with known specimen type; N=972,673).

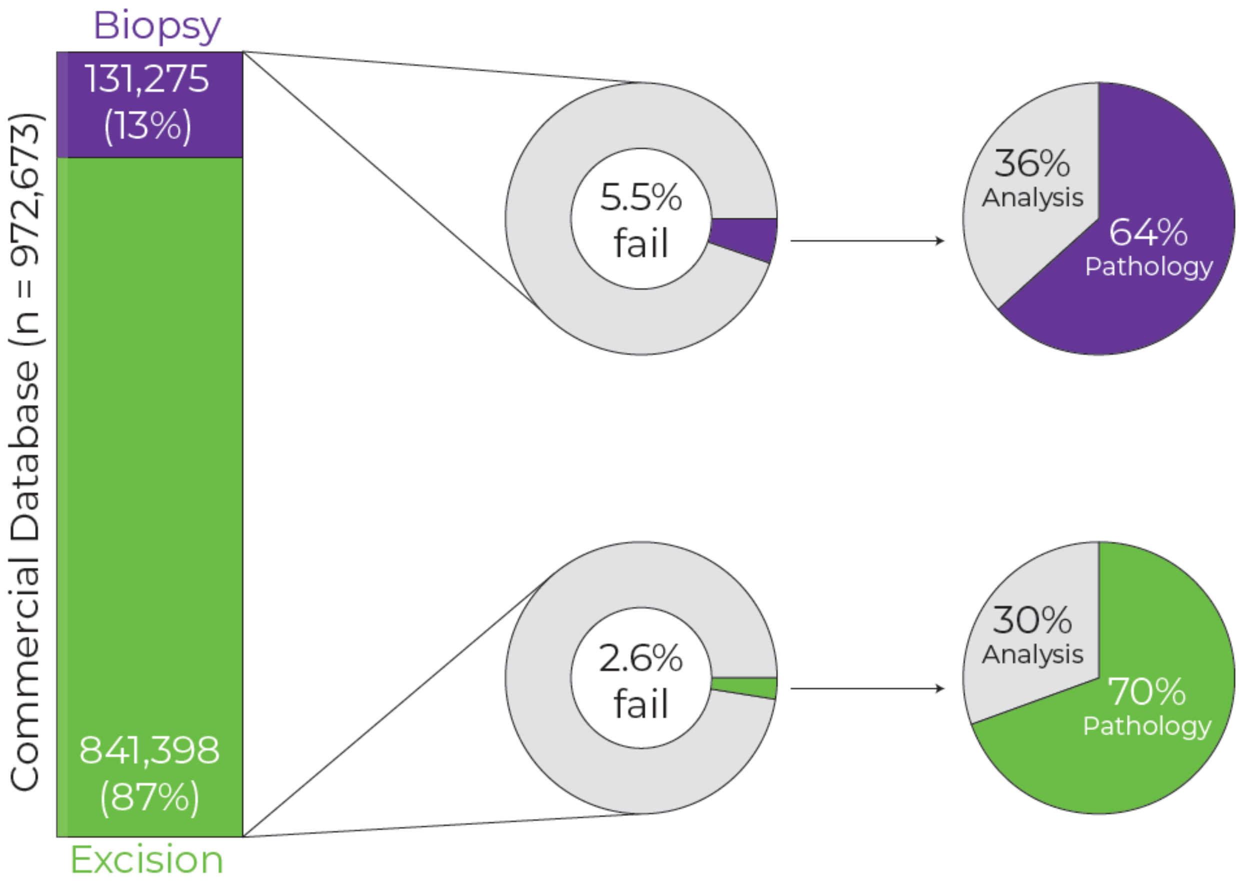

Supplementary Figure 2. Distribution of Recurrence Score results by age and specimen type. Vertical dashed lines indicate the RS 11-25 group (N=919,701).

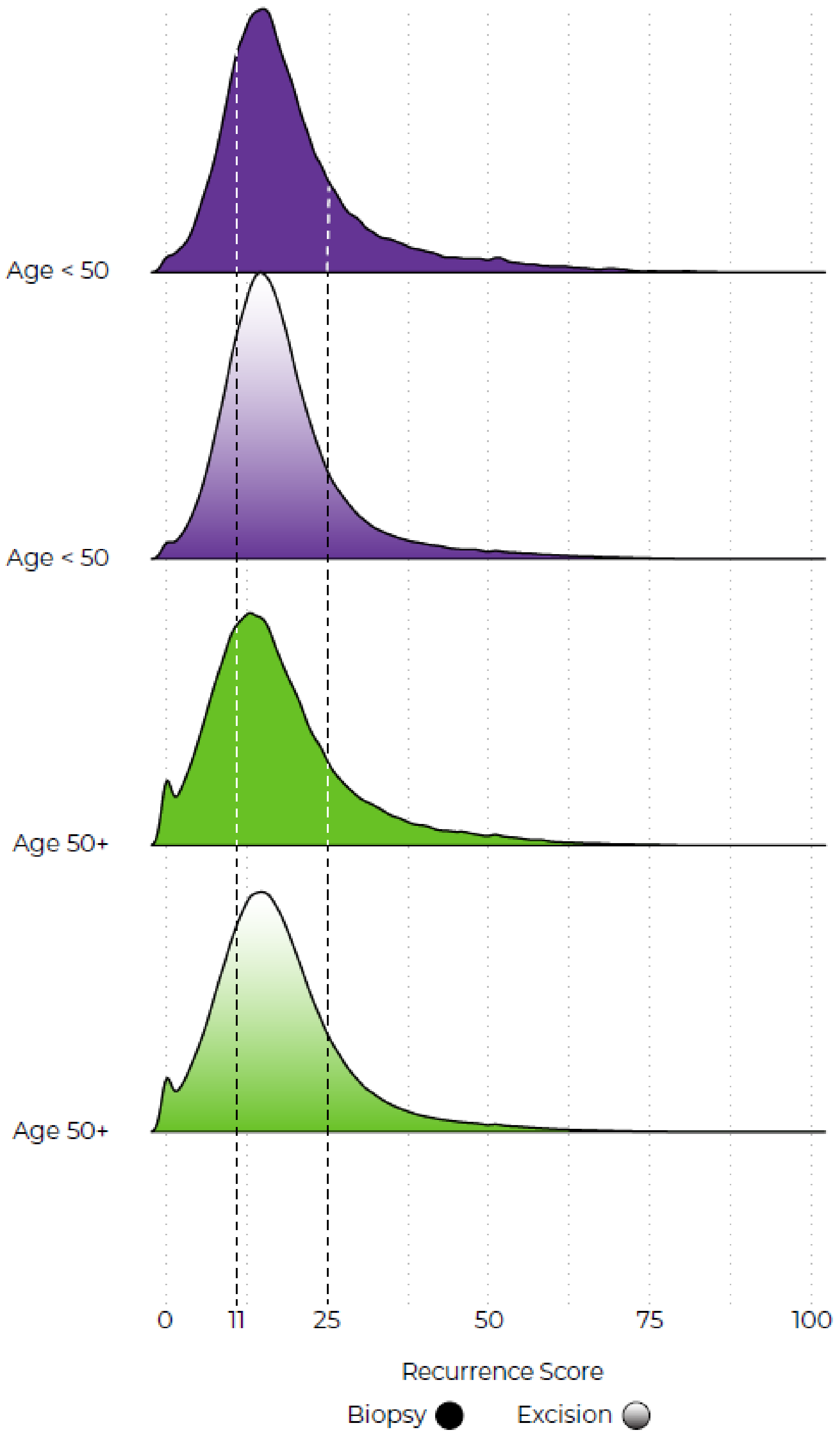

Supplementary Figure 3. Distribution of Recurrence Score results by nodal status. Vertical dashed lines indicate the RS 11-25 group (N=919,701).

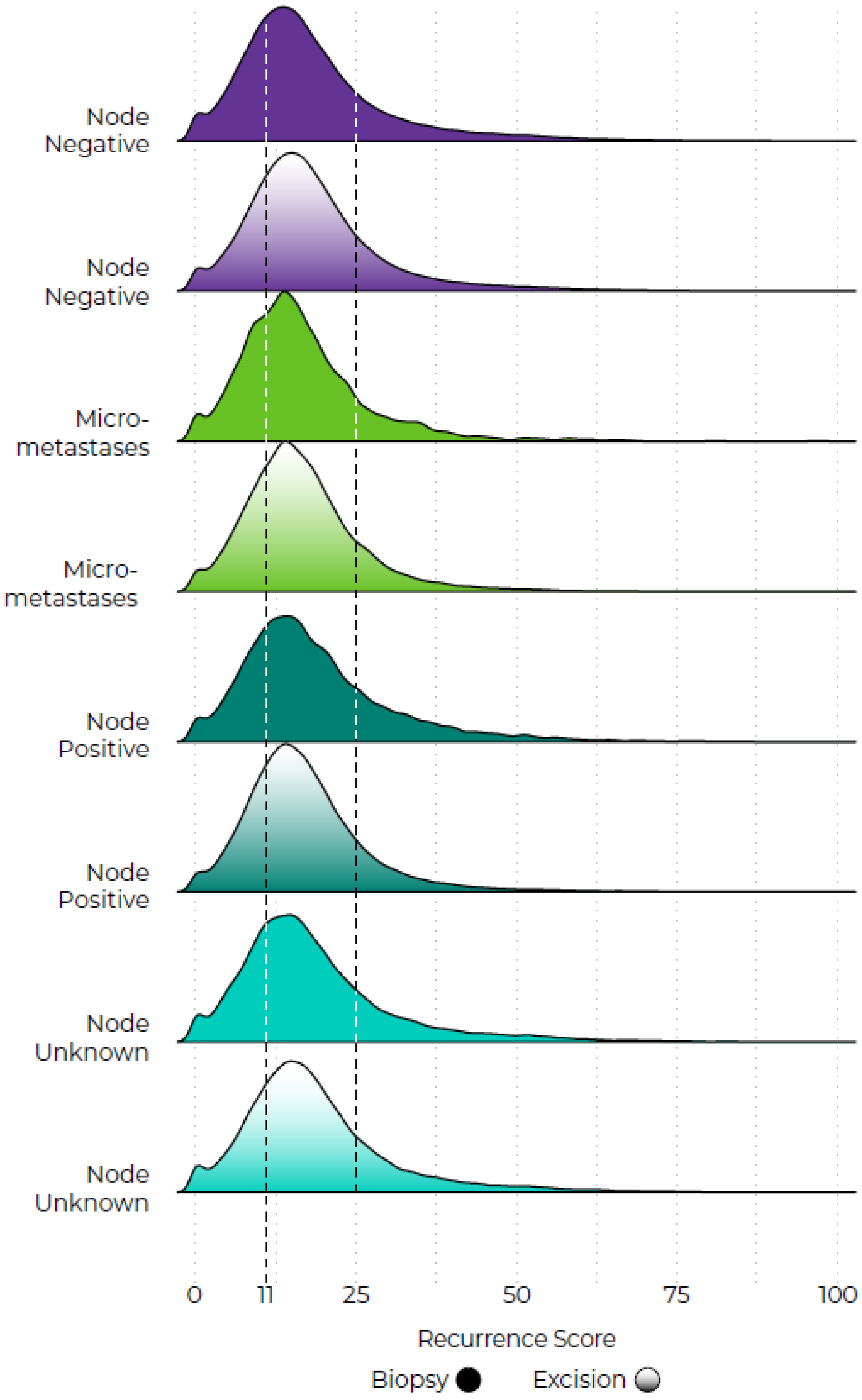

Supplementary Figure 4. Distribution of Recurrence Score groups by age and nodal status (N=919,701).

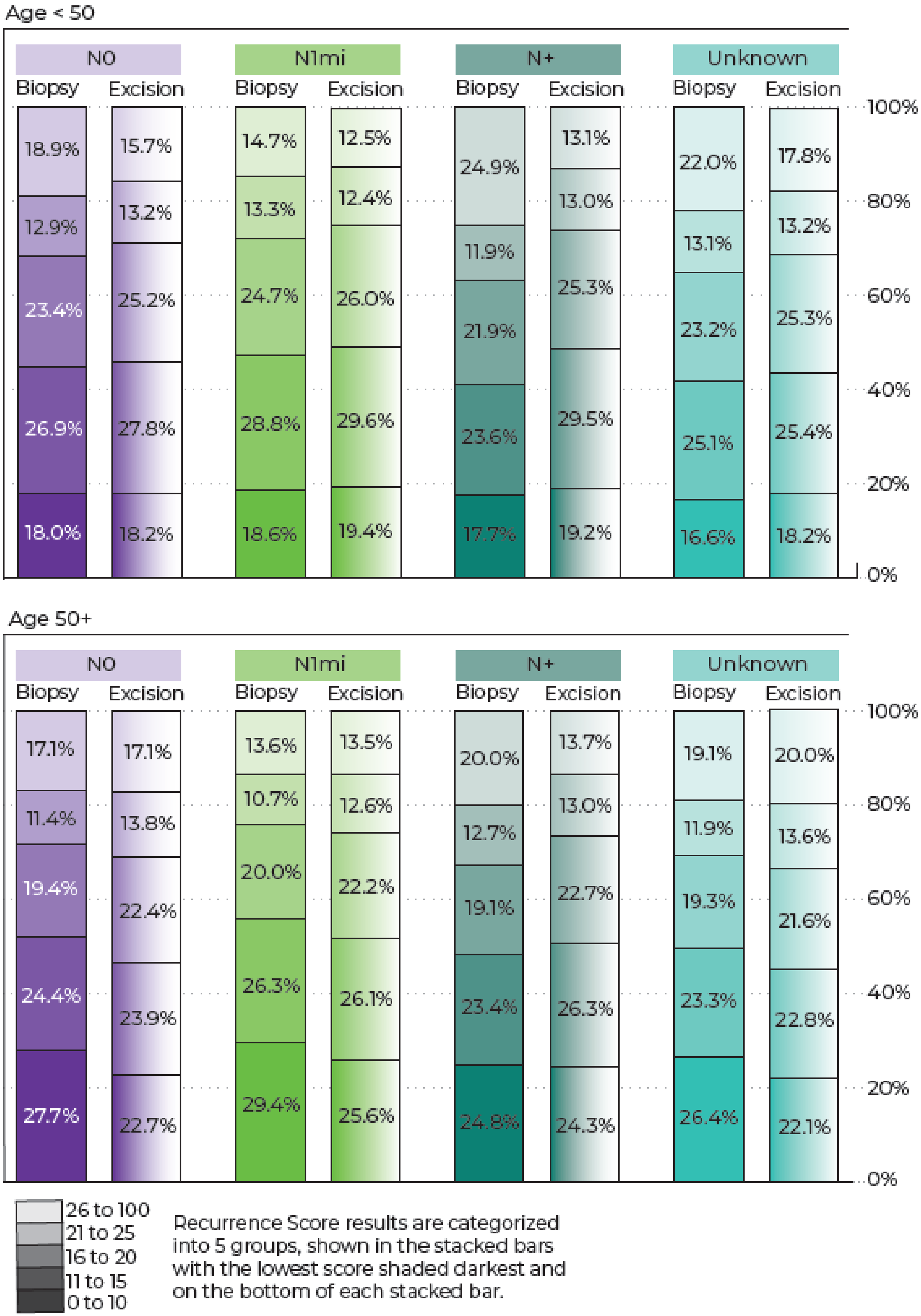

Supplement: Supplementary file 1 — Supporting information [file JSO-122-611-s001.pdf]
